# Do Piperonyl Butoxide Long-Lasting Insecticide Treated Nets Provide Additional Protection Against Malaria Infections Compared with Conventional Nets in an Operational Setting in Western Kenya?

**DOI:** 10.4269/ajtmh.25-0211

**Published:** 2025-11-18

**Authors:** Karla Rascón-García, Zena Lapp, Christine F. Markwalter, Emmah Kimachas, Lucy Abel, Andrew Obala, Steve M. Taylor, Wendy Prudhomme O’Meara, Judith Nekesa Mangeni

**Affiliations:** ^1^ Duke Global Health Institute, Duke University, Durham, North Carolina;; ^2^ Academic Model Providing Access to Health Care, Eldoret, Kenya;; ^3^ School of Medicine, College of Health Sciences, Moi University, Eldoret, Kenya;; ^4^ Division of Infectious Diseases, Duke University School of Medicine, Durham, North Carolina;; ^5^ School of Public Health, College of Health Sciences, Moi University, Eldoret, Kenya

## Abstract

Malaria control in sub-Saharan Africa has stagnated despite widespread adoption of control measures such as long-lasting insecticidal nets (LLINs). Progress has stalled, in part, because of pyrethroid insecticide resistance, driving the need for retooling to increase the effectiveness of bed nets. Consequently, LLINs have been treated with the chemical synergist piperonyl butoxide (PBO). Piperonyl butoxide LLINs have been shown to be efficacious in controlled settings; however, their effectiveness in real**-**world settings warrants investigation. In Bungoma County, Western Kenya, a cohort of 768 participants was followed from June 2017 to December 2023 via active and passive surveillance. Household visits were conducted monthly, during which LLIN use for nets distributed in 2017 and 2021 was recorded, and symptomatic malaria cases were identified using rapid diagnostic tests (RDTs). The comparative effectiveness of PBO versus conventional LLINs was assessed in terms of malaria infections. A multilevel logistic regression model was fit with monthly RDT results as the dependent variable. The study results indicate that PBO LLINs provide greater protection against malaria at the individual level than conventional LLINs (odds ratio: 0.70; 95% CI: 0.47–1.03), although the findings were not statistically significant. The added protection against malaria infections provided by PBO LLINs compared with conventional LLINs observed in the current study aligns with findings from most previous studies, although this finding was not statistically significant. In areas with documented pyrethroid resistance, the use of LLINs with an added synergist, such as PBO, can provide additional protection against malaria infections (compared with pyrethroid**-**only LLINs) and should be considered for scaled-up scenarios despite the additional cost.

## INTRODUCTION

Long-lasting insecticidal nets (LLINs) are the cornerstone of malaria prevention efforts across sub-Saharan Africa. Since 2004, more than 2 billion LLINs have been distributed globally.^1^^,^^2^ As a result, household ownership of LLINs in sub-Saharan Africa rose from 5% to a peak of 75% by 2017,^3^ contributing to a more than 40% decline in malaria incidence and deaths across the continent.^1^^,^^2^ However, since 2015, global malaria cases have plateaued, with some regions experiencing resurgence, including areas where control efforts had previously been successful.^4^^–^^6^

Malaria control has plateaued for several reasons, including stagnating financial support. In 2018, for example, there was a funding gap of ∼5.0 billion US dollars compared with the estimated global amount necessary to maintain progress toward malaria control targets.^3^^,^^7^^,^^8^ The situation is further complicated by the spread of vector resistance to pyrethroid insecticides, which has likely contributed to a resurgence of malaria in some regions.^2^^,^^3^^,^^6^^,^^9^ Pyrethroids have traditionally been used to impregnate bed nets owing to their favorable safety profile, relatively lower cost, and rapid insecticidal activity against mosquitoes with minimal harm to humans.^2^^,^^10^^,^^11^

The rapid and widespread pyrethroid resistance present in most parts of Africa and Asia has driven the need for new strategies or retooling of existing ones to overcome this challenge.^12^^–^^16^ In response, next-generation LLINs have been designed to combat insecticide-resistant, host-seeking mosquitoes by killing or sterilizing them or by reducing their ability to reproduce. Next-generation nets intended to kill resistant mosquitoes typically involve a combination of pyrethroid insecticides and additional active ingredients. One such type includes nets treated with both pyrethroids and the synergist piperonyl butoxide (PBO), which inhibits mosquito metabolic enzymes responsible for detoxifying insecticides, thereby enhancing their effectiveness. Another category, known as dual-active ingredient nets, combines pyrethroids with non-pyrethroid insecticides. For example, nets that include both alpha-cypermethrin (a pyrethroid) and chlorfenapyr (a pyrrole insecticide) have already been tested and shown promising entomological and epidemiological impacts.

The second type of next-generation LLINs is designed to sterilize resistant mosquitoes or reduce their fecundity. These nets also involve dual active ingredients, typically a pyrethroid and an insect growth regulator, such as pyriproxyfen. Pyriproxyfen disrupts mosquito reproduction and development, effectively sterilizing female mosquitoes and thereby limiting their population growth.^17^^–^^21^ Findings from multiple field studies conducted to assess the efficacy of next-generation LLINs against malaria indicators in areas with documented pyrethroid resistance have revealed significantly greater effectiveness in reducing both malaria infections and malaria vectors compared with conventional LLINs.^22^^–^^26^ The WHO now recommends their use in areas where pyrethroid resistance is either confirmed or is at an intermediate level and linked, at least partially, to monooxygenase-based resistance mechanisms.^27^ Next-generation LLINs with an incorporated synergist PBO, which works by inhibiting the P450 detoxification enzymes responsible for mosquitoes’ metabolic resistance and thereby restoring mosquito susceptibility to pyrethroids, have recently been deployed for use. Although PBO LLINs have exhibited promising results in primarily controlled environments, limited research has been conducted in real-world settings, and their effectiveness in these contexts is still being assessed.

Despite their proven effectiveness, however, PBO LLINs cost at least an additional dollar per LLIN delivered compared with conventional LLINs,^28^ which has limited their widespread distribution to areas experiencing a rise in insecticide resistance. In Kenya, the first deployment of PBO-impregnated LLINs took place in July 2021, targeting three lake-endemic counties through a pilot project. Bungoma County, Kenya, was among those regions that received PBO LLINs. In July 2024, the Kenya National Malaria Control Program (NMCP) coordinated a second deployment of these next-generation bed nets in the same counties. Before the introduction of PBO LLINs in Kenya, conventional LLINs were primarily distributed through antenatal clinics and child welfare clinics, targeting pregnant women and children under 1 year of age, mainly in Western Kenya, in 2006. In subsequent years (specifically, 2010 and 2014), the distribution was expanded to cover the general population. Initially, these nets required regular retreatment by households. Every 3 months, families would dip the nets into a solution mixed with a pyrethroid-based tablet. However, this changed in 2017 with the introduction of LLINs. These new LLINs were pretreated with pyrethroids designed to remain effective throughout the net’s entire lifespan (∼3 years), eliminating the need for periodic retreatment by users. The brand name of the LLINs distributed in 2017 was Olyset (Sumitomo Chemical, Tokyo, Japan).^29^

Since 2017, a community-based cohort has been monitored in a setting with seasonal *Plasmodium falciparum* transmission in Bungoma County, Kenya. In 2017, the NMCP distributed conventional LLINs (Olyset brand) for the first time. Distribution was performed through mass net campaigns, and members of the study cohort in Bungoma County were among the recipients. Before the distribution process, a census registration of households and their members was conducted by the NMCP team; collection was performed at designated collection points, mainly at nearby hospitals or schools. For each household, a bed net was provided for every two people. However, because of the growing problem of pyrethroid resistance, next-generation LLINs treated with a synergist PBO were introduced in 2021. As noted, Bungoma County was one of the three lake-endemic counties selected for the initial pilot PBO LLIN distribution. A similar process to the one used in 2017 was followed for the registration and distribution of the bed nets. Since the NMCP distributed conventional LLINs in 2017, data on net usage, malaria infections, and mosquito populations have been collected. This longitudinal data collection, coupled with the recent deployment of PBO LLINs, provides a unique opportunity to assess their real-world effectiveness compared with conventional LLINs. The study authors hypothesized that compared with conventional LLINs, PBO LLINs would provide a significantly greater protective effect against malaria infections at the individual level. Understanding whether PBO LLINs offer a significant advantage over their predecessors will be crucial for determining the return on investment, particularly in regions where malaria transmission remains stubbornly high.

## MATERIALS AND METHODS

### Study population, setting, and design.

The present study was conducted in Bungoma County, one of Kenya’s high-malaria burden regions, which is also classified as a lake-endemic area. The incidence of malaria infections in Bungoma County in 2019 was estimated at 200 cases per 1,000 population, higher than the national average of 125.92 cases per 1,000 population.^30^ *Anopheles gambiae* s.l. is the most predominant malaria mosquito species (up to 75% of the mosquito population) in the region, with a sporozoite rate of 9% for indoor resting mosquitoes and 4% for outdoor resting mosquitoes.^31^

The current study is part of a larger longitudinal cohort study launched in June 2017, aimed at understanding malaria transmission dynamics in the region to inform effective interventions. Continuous monitoring of malaria indicators, including mosquito population densities and malaria infections, has been ongoing, with weekly mosquito collections and monthly and annual surveys conducted in five high-malaria burden villages that comprise the study cohort. These villages are part of the former Webuye Health and Demographic Surveillance Sites (HDSSs) established in Bungoma County in 2007 to provide reliable demographic, health, and economic data for research and planning. Details of the full radial sampling scheme have been described previously.^32^

A subset of households within the former HDSSs was enrolled in this cohort using an open cohort design, starting with randomly selected index households and followed by the selection of neighboring households until 12 households per village were enrolled in natural clusters. In 2020, the present study was expanded to include two additional villages, increasing the total to five. Over the course of the study period, whenever households opted out of the study, neighboring households were contacted to replace them and maintain a 12-household-per-village sampling rate. These villages were chosen because of their high malaria transmission burden. All household members over the age of 1 year who regularly slept in the household were enrolled.

The villages are located within Webuye East and Webuye West subcounties in Bungoma County, ∼50 kilometers from the Uganda border. The region’s primary economic activity is small-scale maize farming, with a few families growing sugarcane commercially. More than 60% of the population lives below the poverty line, with limited access to basic amenities such as water and electricity. Malaria transmission in this area is perennial, peaking after the rains in May and June.

### Study procedures.

#### Active surveillance for insecticide-treated net use.

Detailed data on household LLIN use was collected monthly, including information about bedtimes, wake times, and LLIN use from the previous night. Conventional LLINs were distributed in 2017, followed by the distribution of PBO LLINs 4 years later, in June 2021. During annual surveys, additional data were collected on sleeping spaces, including the presence of nets (whether PBO or conventional LLINs) and household members occupying each space. The condition of each net was visually inspected, and net-specific information was recorded, including the number and size of holes, age (the date when the net was acquired), source, and how the net was being used (hung correctly, permanently hung, or removed daily).

Moreover, during these monthly visits, participants were asked whether they had experienced malaria-like symptoms in the month preceding the monthly visit and whether a rapid diagnostic test (RDT) had been performed. If an RDT was conducted, participants were further asked whether the RDT results were positive or negative and whether the test was conducted by the study team or at another health facility. Monthly and annual visit data were collected using separate REDCap survey tools (Vanderbilt University, Nashville, TN).

#### Passive surveillance of malaria episodes.

Cohort members were encouraged to request malaria testing, which was conducted by the study team, whenever someone in their household had a fever. During these sick visits, presenting symptoms were documented, and a malaria RDT was performed. Moreover, a malaria slide was also prepared for confirmatory microscopy. If the test result was positive, the patient received antimalarial medication (artemether***–***lumefantrine) at no cost from a pharmacy within the cohort study area. Patients with severe cases or persistent fevers were referred to the hospital. Those with negative cases and lingering symptoms were referred to the nearest health facility. Monthly follow-ups were used to track whether patients took their medication, completed the dosage, and took further steps. Sick visit records were collected using a REDCap survey tool that was distinct from the monthly and annual survey tools.

#### Collection of entomology indicators.

On the same day of each week, households were visited between 6 am and 7 am, and mosquito captures were performed via vacuum aspiration. Participants were always instructed to leave doors and windows shut before the arrival of the study team for mosquito aspirations. Captured mosquitoes were stored in collection cups within ice-packed cool boxes and were subsequently transported to the laboratory, where the sex and genus of captured mosquitoes were recorded, and female mosquitoes were graded according to abdominal status. Magnified images of female *Anopheles* mosquitoes were captured for post hoc speciation. These mosquito capture data facilitated the study of mosquito density trends before and after the introduction of PBO LLINs, as well as changes in species (Culex versus *Anopheles*) populations over time in relation to LLIN use.

### Measurements.

#### Exposure and outcome variable.

The main exposure was monthly reported bed net use in the month preceding an RDT. The exposure variable was categorical with three levels: slept under a conventional LLIN (reference level), did not sleep under a LLIN, or slept under a PBO LLIN. More specifically, participants were asked whether they had slept under a bed net the night before their visit, and we assumed that this response reflected each participant’s most likely behavior in the month preceding the sick visit.

The primary outcome of interest was whether an individual had a positive or negative malaria RDT result. Rapid diagnostic test results were observed through either passive surveillance of sick visits conducted by the study team or the active surveillance of self-reported RDT results during monthly visits.

#### Model covariates.

Demographic variables considered in the model included sex, the participant’s age at the time of the visit (<5, 5 to 15, or >15 years), village, and study year. Age at the time of visit was estimated by comparing each participant’s birth year with the year in which the monthly survey or sick visit was conducted.

Net-specific characteristics, including net age in months and the presence of holes in the net, were also included as covariates. During annual surveys, participants reported the date they acquired a net. The age of the net was calculated as the difference in months between the date of their monthly or sick visit and the reported date of acquisition. Moreover, nets were visually inspected by the study team during annual visits and appraised for the presence of holes. Although the presence or absence of holes was recorded, the quantity and size of the observed holes were not considered.

Lastly, temporally varying high- and low-transmission months were controlled for in the analysis. High-transmission months were either defined as 1) the historically high-transmission months of May to July or 2) the mean number of female *Anopheles* collected per village in a month, where a monthly count >45 (85th percentile cutoff value) was considered a high-transmission month.

## STATISTICAL ANALYSES

Multilevel logistic regression was used to assess differences in the risk of malaria infection between individuals with different types of bed net use (those who did not sleep under a LLIN, those who slept under a PBO LLIN, and those who slept under a conventional LLIN). Two models were fit, one with and one without a random slope for annual time steps, where the outcome in both models was binarized (positive or negative) RDT results in a given month.

Descriptive statistics of sociodemographic variables, including frequencies and proportions, were summarized for participants across the entire study period. Participant age at the time of visit was visually described using a histogram distribution. Moreover, descriptive statistics related to main exposure events (did not sleep under a LLIN, slept under a PBO LLIN, or slept under a conventional LLIN) were compared across all covariates considered in the analyses.

Participants who either 1) requested a sick visit and had an RDT conducted by the study team or 2) self-reported results (during a monthly visit) of an RDT conducted at a different facility, made up the subset of observations that constituted the input data analyzed in the present study. A comparison of observations from participants who requested an RDT with those from participants who neither requested nor reported having taken one is provided in Supplemental Table 1. This comparison was conducted to assess the representativeness of the study sample because the attributes and behaviors of this subset may not reflect the larger population. Moreover, the demographic characteristics of participants in relation to bed net use are provided in Supplemental Table 2. All analyses were conducted using R version 4.3.1 (R Foundation, Vienna, Austria), with the primary packages used including *lme4*, *performance*, *mlmhelpr*, and *ggplot2*.^33^^–^^36^

### Multilevel logistic regression.

Three models were fit with random intercepts for households and participants:

- **Model 0**: Null model, includes the study year as a fixed variable (baseline model);
- **Model 1**: Full model with individual and community-level factors (no random slope);
- **Model 2**: Full model with individual and community-level factors (random slope for study year).

The final model (Model 2), which included predictors from all three levels (Supplemental Figure 1), was used to examine the effects of individual- and community-level factors on the risk of malaria infection as measured using positive RDT results. By including the study year as a random effect (rather than a fixed effect), individual-level infection risks could vary from year to year. Adjusted odds ratios (ORs) and their 95% CIs were calculated using the Wald test to describe the strength of association between outcome and predictors.

### Multicollinearity and missing data.

The variance inflation factor (VIF) was used to test for multicollinearity among covariates; predictors with a VIF value less than 4 indicated no inter-variable multicollinearity.

The method for handling missing data depended on the context of missingness: if bed net use data were missing for the month before an RDT report, bed net use data from the month of the positive RDT were used instead. Records in which bed net type (conventional LLIN versus PBO LLIN) could not be determined were dropped because they were noninformative (3.9% of all records). The bed net type could not be determined if the date when the bed net was acquired was also missing after June 2021 because all nets with acquisition dates before the June 2021 distribution of PBO nets were assumed to have been conventional LLINs.

Net-specific predictors (e.g., net age, the presence of holes in the net, how often participants washed their bed nets, etc.) were considered covariates during model building because the characteristics of the hanged LLINs could still confound estimates. However, missingness across these variables had to be addressed. Net washing frequency could not be considered a covariate because of its high rate of missingness (39.1%). Although net age (12.4% missing) and net integrity (net with or without holes) exhibited modest levels of missingness (19.3%), these variables were retained, and sensitivity analyses were conducted to evaluate the influence of these predictors.

### Sensitivity analyses.

Sensitivity analyses were conducted to account for the three potential sets of bias or misclassification. The first set consisted of net-specific variables (i.e., the date the net was acquired and the presence or absence of holes on the net), which were characteristics obtained from the annual survey; however, issues with missing or incomplete data were encountered. Net washing frequency was dropped from consideration because of its high rate of missingness (39.1%). Data on the date the net was acquired, used to estimate the net’s age in months, and whether the net was intact or had holes were also missing, with missingness rates of 12.4% and 19.3%, respectively. To assess the impact of including these net-specific variables in the model, sensitivity analyses were conducted by comparing a model that included them with one that did not, and the direction of the effects was reported.

Second, when defining the composite variable of high transmission, high-transmission months (May, June, and July) and the 85th percentile of mosquito abundance were used to define months with a high risk of malaria transmission. Sensitivity analyses were conducted to evaluate the effect of using two alternative approaches: 1) using only fixed months of May, June, and July as high-transmission months or 2) using different mosquito abundance thresholds (80th and 90th percentiles) as alternative cutoffs.

To account for any potential bias that could arise in this longitudinal analysis from including observations before PBO nets were distributed, the model was tested in four scenarios: 1) the full time window (June 2017 through December 2023), with net-specific covariates included, 2) the full time window without net-specific covariates, 3) a shortened time window that only included observations starting from the month when PBO nets were distributed (June 2021 through December 2023), with net-specific covariates included, and 4) this shortened time window without net-specific covariates.

Finally, because of observed differences in the net type used between individuals who requested an RDT (analytic population) and those who did not, a weighted model was considered, and its estimates were compared with those of the final unweighted model. Individuals who had an RDT performed reported having slept under a PBO net 12.8% of the time, compared with 21% among those who slept under a conventional LLIN. This underrepresentation of PBO net use could bias results toward the null; consequently, a weight model adjusting for sampling rates was explored.

### Post hoc power analysis.

The power of the present study was evaluated by conducting a post hoc power analysis for a multilevel process model and investigating the causal process within-persons as described by Bolger and Laurenceau^37^ using the simr package in R.^38^ Using an alpha of 0.05, 1,000 simulations were run on a full model to estimate the present study’s power to detect 1) differences between PBO and conventional nets (primary objective) and 2) differences between not sleeping under a LLIN and sleeping under conventional nets.

## RESULTS

### Demographic characteristics.

**Figure 1.**
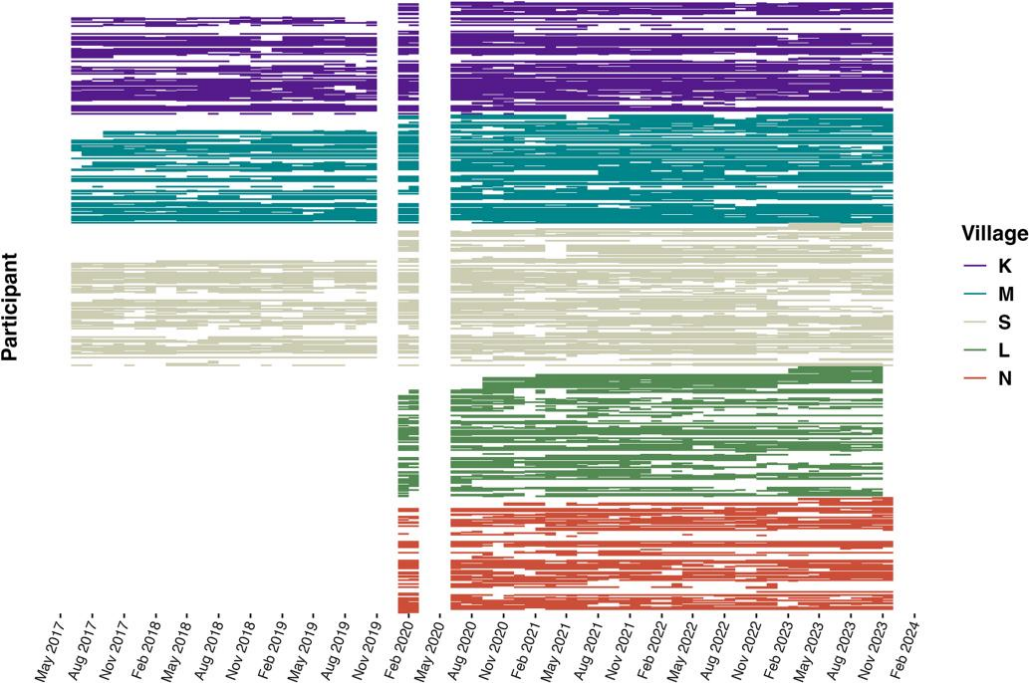
Participants’ monthly and sick visits over time. Each participant is represented by a row within their village. Cohort activities stopped for 2 months in April and May 2020. Villages L and N joined the cohort in 2020, and village L left the cohort in December 2023.

The initial cohort comprised 280 participants in June 2017; however, after the expansion from three to five villages in 2020, the number increased to more than 750 by the end of the study period in December 2023 (Figure 1). Every household in the study owned at least one LLIN.

A total of 768 participants from five villages and 85 households in Western Kenya were followed via active monthly surveillance for up to 76 months. Between June 12, 2017 and December 12, 2023, a total of 23,477 monthly or sick visits were recorded among these participants. Participant ages ranged from 1 to 101 years at the time of visit (Supplemental Figure 2), with 51.3% being 18 years of age or younger at the end of the study in 2023. Of all 768 participants enrolled, 512 (66.7%) requested a sick visit during the study period. As it relates to the study outcome (RDT results), ∼18% of observed outcomes were self-reported (although confirmation was performed by checking the clinical booklets used at the facilities to ensure the presence of a true infection), whereas 78% were obtained through sick visits performed by the study team via RDTs, with positivity confirmed using microscopy. Demographic characteristics of this analytic population, including participants who either had an RDT performed by the study team or reported RDT results conducted by another facility, are presented in Table 1.

**Table 1:** Sociodemographic descriptive statistics of the analytic population (*N* = 512)

Characteristic	*n* (%)
Village	
Village K	107 (20.9)
Village L	77 (15.0)
Village M	116 (22.7)
Village N	78 (15.2)
Village S	134 (26.2)
Sex	
Female	284 (55.5)
Male	228 (44.5)
Age in 2023 (years)	
<5	51 (10.0)
5 to 15	170 (33.2)
>15	291 (56.8)
Education	
No education	93 (18.2)
Primary—some	263 (51.4)
Primary—complete	51 (10.0)
Secondary—some	54 (10.5)
Secondary—complete	38 (7.4)
Tertiary/university	13 (2.5)
Employment	
Not used	70 (13.7)
Used—formal	17 (3.2)
Self-used—business	34 (6.6)
Retired	1 (0.2)
Other (i.e., student or unable to work)	390 (76.2)

**Figure 2.**
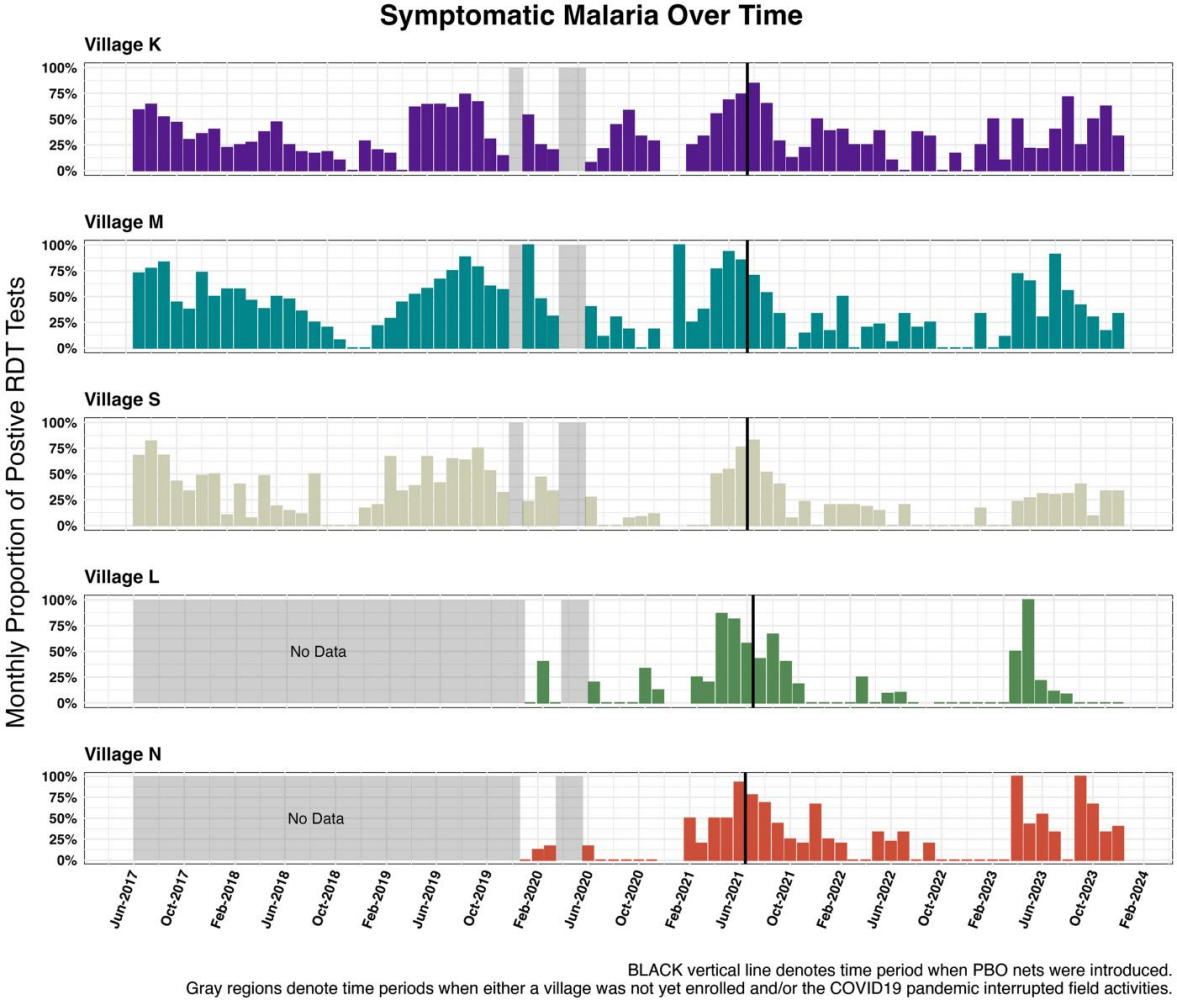
Symptomatic malaria over time: proportion of positive rapid diagnostic test results from monthly and sick visits.

Of these 23,477 records, 3,431 (14.6%) involved visits from participants who either requested a sick visit and had an RDT conducted by the study team or attended a regular monthly visit in which they reported having had an RDT performed during the previous month at a health facility (Table 2). Of these 3,431 visits, 1,443 (42%) involved positive results across 512 participants who took an RDT. The monthly prevalence of symptomatic malaria presented seasonal variations over time (Figure 2). Village M reported the highest malaria prevalence (50.5%) among participants who took an RDT.

**Table 2:** Descriptive statistics of the main exposure (bed net use) across the analytic population

Mosquito Net Use Reports from Monthly Surveys	Overall *N* = 3,431*	Conventional LLIN *n* = 1,956*	PBO Net *n* = 439*	Did Not Sleep Under Net *n* = 1,036*
Village				
K	1,019 (29.7%)	713 (36.5%)	114 (26.0%)	192 (18.5%)
L	205 (6.0%)	108 (5.5%)	25 (5.7%)	72 (6.9%)
M	972 (28.3%)	565 (28.9%)	144 (32.8%)	263 (25.4%)
N	250 (7.3%)	72 (3.7%)	61 (13.9%)	117 (11.3%)
S	985 (28.7%)	498 (25.5%)	95 (21.6%)	392 (37.8%)
Sex				
Female	1,930 (56.3%)	1,161 (59.4%)	253 (57.6%)	516 (49.8%)
Male	1,501 (43.7%)	795 (40.6%)	186 (42.4%)	520 (50.2%)
Age at monthly of visit (years)				
<5	481 (14.0%)	325 (16.6%)	68 (15.5%)	88 (8.5%)
5 to 15	1,481 (43.2%)	734 (37.5%)	168 (38.3%)	579 (55.9%)
>15	1,469 (42.8%)	897 (45.9%)	203 (46.2%)	369 (35.6%)
Net age (months)	21 (10, 32)	23 (12, 35)	13 (5, 23)	21 (11, 34)
Missing or N/A	426	104	0	322
Net holes				
Net had at least 1 hole	1,663 (60.2%)	1,127 (63.5%)	205 (47.8%)	331 (59.2%)
Net intact	1,100 (39.8%)	648 (36.5%)	224 (52.2%)	228 (40.8%)
Missing or N/A	668	181	10	477
Malaria season				
Low-transmission season	1,898 (55.3%)	1,123 (57.4%)	293 (66.7%)	482 (46.5%)
High-transmission season	1,533 (44.7%)	833 (42.6%)	146 (33.3%)	554 (53.5%)

LLIN = long-lasting insecticidal net.

* Person-months, *n* (%); median (interquartile range).

General bed net use, independent of the type of net, was not statistically significant (*P* = 0.20) between participants who took an RDT and those who did not (Supplemental Table 1). This finding suggests that the analytic population was unlikely to be biased, as they did not practice significantly different sleeping behaviors from participants who did not take an RDT. A significant difference, however, was observed between net types (PBO versus conventional), with individuals who requested an RDT being more likely to have slept under a conventional mosquito net. Approximately 84% of participants reported sleeping under a bed net at least once during this study. During visits when an RDT was requested, PBO net use was lower (12.8% of visits) than the higher compliance rate for conventional nets (57% of visits).

**Figure 3.**
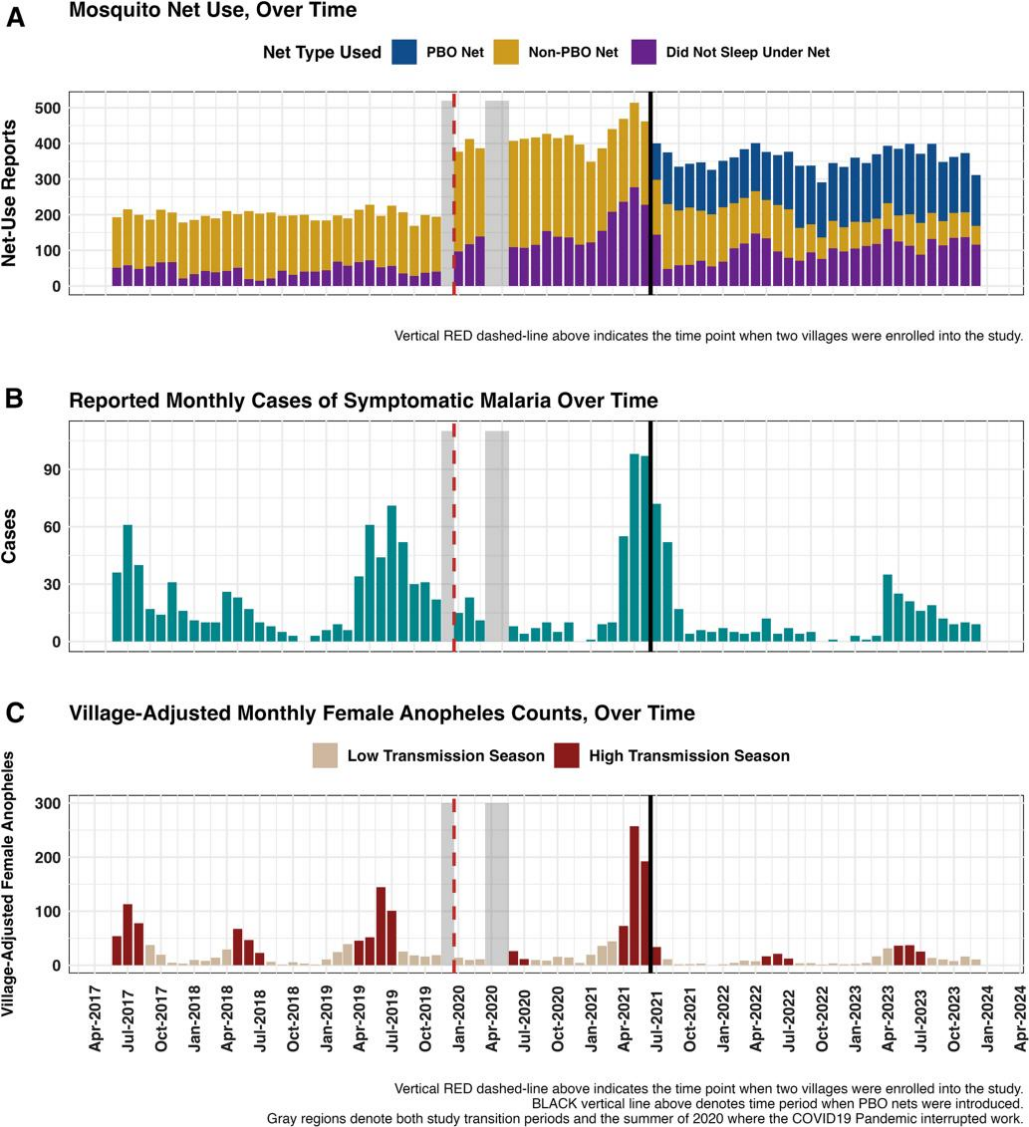
Temporal trends for (**A**) net use (main exposure), (**B**) the prevalence of symptomatic malaria (rapid diagnostic test [RDT] results, outcome of interest) over the complete study period, and (**C**) malaria high-transmission seasons as defined by monthly anopheles captures and historically high transmission months (see the methods for more details). (**A**) Total monthly reports of participants who either slept under a conventional long-lasting insecticidal net (LLIN), slept under a piperonyl butoxide LLIN, or reported having not slept under a mosquito net. (**B**) Monthly prevalence of symptomatic malaria across all villages, as determined by reported RDT results. (**C**) Months classified as high-transmission versus low-transmission using a composite definition where the 85th percentile of *Anopheles* captures (40+ mosquitoes) and historically high-transmission months (May, June, and July) are collectively defined high-transmission months.

Despite the distribution of PBO bed nets in June 2021, conventional LLIN use continued because not all participants adopted the newly distributed nets (Figure 3A). As expected, seasonal variation was observed in both symptomatic malaria episodes (Figure 3B) and female anopheles mosquito abundance over time (Figure 3C).

To address the main research question of whether PBO bed nets offer additional protection against malaria infections compared with conventional bed nets, malaria infection ORs were estimated in multilevel models, both with and without a random slope for time (Table 3).

**Table 3:** Multilevel model results for full study time window (June 2017–December 2023)

	Multilevel Models, OR (95% CI)		
	Model 0	Model 1	Model 2
Model Covariate	Baseline Model, Includes Time	Full Model, No Random Slope	Longitudinal Model, Includes Random Slope
Study year	**0.91 (0.87–0.95)**	**0.90 (0.83–0.96)**	**0.89 (0.81–0.96)**
Conventional bed net		Ref	Ref
Did not sleep under bed net		**1.73 (1.34–2.23)**	**1.70 (1.31–2.21)**
PBO bed net		0.72 (0.50–1.05)	0.70 (0.47–1.03)
Village			
K		Ref	Ref
L		**0.49 (0.27–0.88)**	**0.50 (0.27–0.93)**
M		1.38 (0.98–1.92)	1.37 (0.94–1.99)
N		1.13 (0.68–1.89)	1.14 (0.67–1.95)
S		0.72 (0.51–1.02)	**0.67 (0.454–0.996)**
Sex			
Female		Ref	Ref
Male		1.15 (0.89–1.49)	1.21 (0.92–1.58)
Age (years)			
<5		Ref	Ref
5 to 15		**1.73 (1.26–2.38)**	**2.12 (1.47–3.06)**
>15		**0.68 (0.48–0.96)**	0.75 (0.51–1.09)
Net age (months)		**1.01 (1.001–1.015)**	**1.01 (1.001–1.016)**
Net intact		Ref	Ref
Net had at least 1 hole		0.96 (0.76–1.21)	0.94 (0.73–1.22)
Low-transmission season		Ref	Ref
High-transmission season		**2.17 (1.81–2.60)**	**2.26 (1.87–2.72)**
SD (intercept, M_ID)	2.548	2.060	3.445
SD (study year, M_ID)			1.326
Cor (intercept ∼ study year, M_ID)			0.429
SD (intercept, hh_id)	1.325	1.085	1.220
Number of observations	3,431	2,691	2,691
R2 Marg.	0.008	0.148	0.159
R2 Cond.	0.231	0.266	0.324
AIC	4,435.2	3,248.1	3,236.4
Deviance	4,427	3,216	3,200

AIC = Akaike information criterion; Cond. = conditional; hh_id = household ID; ICC = intraclass correlation coefficient; Marg. = marginal; M_ID = participant ID; OR = odds ratio; PBO = piperonyl butoxide. Bolding indicates significant OR, with a significance level of *P* <0.05.

After controlling for all factors, PBO bed nets were found to offer additional protection against malaria infections compared with conventional LLINs, although this finding was not statistically significant (OR: 0.72; 95% CI: 0.50–1.05). Because bed net use was studied over time, increasing net age was associated with an increased individual risk of malaria, suggesting a possible decline in LLIN effectiveness over time (OR: 1.01; 95% CI: 1.001–1.016). Associations between factors known to influence bed net use and, therefore, the distribution of malaria infections, such as age, were also examined. There was a significant association between age groups at the time of the visit, with school-aged children (ages 5 to 15 years) estimating a higher odds of malaria infection when compared with children 5 years of age and under (OR: 2.12; 95% CI: 1.47–3.06).

Before PBO LLIN distribution, the density of female *Anopheles* mosquitoes over time exhibited seasonal peaks that coincided with high malaria transmission months. After the introduction of PBO nets in June 2021, a significant drop in monthly female *Anopheles* captures was observed (Supplemental Figures 4 and 5). Monthly female *Culex* mosquito densities, however, did not significantly differ after the introduction of PBO LLINs, suggesting that the female *Anopheles* population declined as a result of PBO LLIN use in the area, rather than any unaccounted environmental phenomena. To compare the differences in mosquito species populations before and after PBO net distribution, Dunn’s test for repeated measures was used to analyze transmission seasons in which the introduction of PBO LLINs caused a significant decline in the female *Anopheles* population (*P* <0.001) while the female *Culex* population exhibited a stable trend (Supplemental Figure 5). Using a VIF cutoff value of 4, no multicollinearity was detected in the final version of Model 2 (Supplemental Table 3) because all variable VIF values were 1.7 or lower.

Sensitivity analyses were conducted using 1) different quantile definitions for a high-transmission season, 2) an evaluation of the effect of including net-specific variables with missing values, and 3) an exploration of the influence of using a full time window (pre-PBO net distribution versus only looking at post-PBO net distribution) across all scenarios. Neither the direction of the effect nor the significance of the observed effect changed (Supplemental Figure 3 and Supplemental Table 4). However, when exploring a weighted model, after adjusting for differences in net type to compensate for PBO LLIN underrepresentation, a weighted model indicated that PBO nets provided significantly greater protection against malaria (OR: 0.52; 95% CI: 0.36–0.76). In contrast, a log-likelihood ratio test indicated that an unweighted model (the final model presented in the results) provided a significantly better fit (*P* <0.001).

Finally, a post hoc power analysis of a full model revealed a high power to detect a true difference between no net use and conventional net use (98%; CI: 92.96–99.76%) but low power to detect a true difference between PBO net use and conventional net use (49%; CI: 38.86–59.20%). Given the low power, the effect of adding more participants or observations (extending the study time period with the same number of participants) on the study power was examined. The results suggest that either 1) a minimum analytic sample size of 800 participants (approximately twice the current analytic sample, *n* = 385 participants) within the current time window or 2) three times more visit records with a sample size of 385 were necessary to achieve a minimum study power of 80% (Supplemental Figure 7).

## DISCUSSION

In this longitudinal cohort, villages in Western Kenya with a historically high malaria infection burden were actively and passively surveyed. With 76 months of follow-up, the odds of malaria infection among individuals with different bed net use practices (no bed net, conventional LLIN, and PBO LLIN) were compared. More specifically, two multilevel logistic regression models, with and without a random slope for annual time-steps, were fitted. The outcome in both models was binary RDT results, either positive or negative, in a given month. Weekly mosquito captures and active monthly surveillance across 85 households over a nearly 6-year study period provided high-resolution data, which were used to retrospectively investigate the effect of PBO bed net use and compare it with conventional bed net use using historical data.

In the present study, the NMCP distributed conventional and PBO LLINs in the community where the cohort is located as part of their malaria prevention strategies. More specifically, the authors evaluated whether PBO LLINs provided additional protection compared with previously used conventional LLINs. In 2017, members of the study cohort received conventional LLINs; by 2021, they received PBO LLINs. Data on LLIN use and malaria indicators were collected from the same population over this entire period, from June 2017 to December 2023.

After controlling for all factors, sleeping under a PBO LLIN provided additional protection against malaria infections compared with a conventional bed net, although the difference was not statistically significant. However, sleeping under a conventional LLIN provided significant protection against malaria infections compared with not using a bed net at all, suggesting that any type of bed net is safer and more effective than none. When in good condition, bed nets serve as a protective barrier, preventing contact between humans and mosquitoes. Previous studies have revealed that even untreated bed nets provide some protection against malaria infections compared with not using a bed net at all because they reduce the human–vector contact.^39^^,^^40^

A nonsignificant protective effect was observed among participants who reported sleeping under a PBO LLIN (versus a conventional LLIN) during the month preceding their RDT. In controlled settings, PBO bed nets have exhibited higher efficacy against malaria indicators than conventional bed nets.^22^^,^^23^ The WHO recommends the use of PBO LLINs in areas with moderate or severe insecticide resistance. Given their high cost, however, PBO LLINs were only distributed to three lake-endemic counties in Kenya. Because this is a modified tool for malaria prevention that is currently being implemented, evaluation is likely ongoing in various settings; therefore, ongoing studies continue to reveal the effectiveness of these bed nets in real-world settings. The present study indicates that PBO bed nets provide additional protection beyond that of conventional bed nets, although the difference is not statistically significant. Furthermore, the results indicate a significant decrease in malaria vectors during the period when PBO LLINs were in use compared with the earlier period when pyrethroid-only bed nets were used. These findings align with those of previous field studies, which have revealed that PBO LLINs provide an additional protective benefit, reducing malaria indices (both infections and mosquito populations) by ∼25–50% or more compared with conventional bed nets.^26^^,^^41^^–^^43^

Net-specific attributes, such as the number of holes in a bed net and the age of the net hanging over a sleeping space, were recorded during annual surveys but were not assessed during monthly visits. Consequently, an inherent assumption regarding net characteristics is that the condition of an LLIN (e.g., the presence or absence of holes) remained true for the time frame between annual visits. In other words, an intact net at the beginning of the year was assumed to be intact until otherwise updated during the subsequent annual visit. Similarly, the net type and its associated age (in months) were assumed to remain unchanged until otherwise updated during any subsequent annual visit. By examining bed net use over time, it was observed that as nets aged, individual risk of malaria increased (OR: 1.01; 95% CI: 1.001–1.016), suggesting a potential decline in LLIN effectiveness over time. Bed net age, how LLINs are handled, and care practices, such as the frequency of washing and the detergents used, could contribute to the loss of effectiveness. Previous studies have also revealed a similar decline in bed net efficacy over time with use.^44^^,^^45^

The study results also suggest that school-aged children (aged 5 to 15 years at the time of visit) had significantly higher odds (OR: 2.26; 95% CI: 1.87–2.72) of symptomatic malaria infection compared with children under 5 years old. This is a common finding in recent studies, which could be because older children are less likely to sleep under bed nets than their younger siblings, who may be given priority.^46^^,^^47^

The key strength of the current study is its longitudinal design, with participants followed up monthly for more than 5 years at monthly intervals. These monthly visits enabled the assessment of participants’ net use behaviors closer to the date of infection. Moreover, although PBO nets were not randomly assigned, considerable heterogeneity in net use was observed, allowing comparison of the risk of malaria pre- and post-PBO net distributions, as well as conventional versus PBO LLIN effectiveness within each time period (Supplemental Figure 6). This extended period enabled a more comparable representation of both conventional LLINs and PBO LLINs (in terms of net age), alongside consistent biomarker collection throughout the study. A full time window was analyzed, both before and after PBO bed nets were distributed for representativeness, as conventional LLINs were distributed in 2017, followed by the distribution of PBO LLINs in June 2021. However, sensitivity analyses revealed no change in the direct effects or their significance, regardless of the time period considered. The analyses were not limited to only include observations from post-PBO bed net distribution because of the inherent limitations identified. A shorter observation time window, combined with an encouraged shift toward the use of newly distributed PBO nets, could have affected the feasibility of detecting a difference between PBO nets and conventional nets. Moreover, with net age as a predictor, limiting analyses to the latter time window would have confounded net age distributions between PBO and conventional nets, in addition to reducing the available records for analysis from 2,691 to 907, weakening the analytic power. Moreover, the high-resolution study data, which also included female *Culex* mosquito densities over the entire study period, facilitated the demonstration of the effect of PBO nets on female *Anopheles* populations by using female *Culex* densities as a reference or control.

Nevertheless, the present study still encountered limitations. The main limitation is related to the study’s power to answer the primary research question. A post hoc power analysis indicated insufficient power to confidently detect a meaningful difference between PBO and conventional net use; therefore, the nonsignificant results of the present study should not be interpreted as conclusive. In a power curve analysis, the effects of two study design variables on the estimated study power were examined. The effect of 1) increasing the number of participants without extending the study period (average of ∼7 visits per participant) or 2) keeping the same sample size (*n* = 385) but increasing the number of monthly visits was assessed. The results suggest that either tripling the number of visits (∼21 visits per participant) or increasing the participant sample size from 385 to 800 participants would be the minimum modifications required to achieve a study power of at least 80% (Supplemental Figure 7). Continuing to observe participants for longer would increase the study’s power to 86%; with the observed time window remaining fixed, a doubled sample size would have strengthened the study’s power. Another limitation of the current study was the type of net hung in households between annual visits. The type of net hung over a sleeping space was captured during annual surveys, whereas monthly surveys captured whether a participant slept under a net in the night before the visit. In the present work, it is assumed that the net type remained constant between annual surveys and did not change between annual visits. Moreover, the main limitation was the missing information for net-specific variables collected during annual surveys, resulting in varying levels of missingness that carried over into the monthly time step analysis. Complete information regarding net washing frequency and more frequent inspection of nets to identify new holes or deformations should be prioritized for collection in future studies. Similarly, regarding the study outcome (RDT results), ∼18% of observed outcomes were self-reported, compared with 78% obtained during sick visits performed by the study team. Self-reported RDT results were confirmed and recorded in clinic booklets, mitigating the possible over-estimation of infections.

## CONCLUSION

Although the findings were not statistically significant, the present study has revealed that PBO LLINs offer additional protection against malaria infection compared with conventional LLINs. However, these bed nets must be handled with care to reduce wear and tear. Additionally, appropriate use by all age groups is required for effective protection. In areas with confirmed pyrethroid resistance, the use of PBO bed nets is more likely to be effective in reducing malaria infections compared with pyrethroid-only bed nets. Moreover, sleeping under a conventional net provided significant protection against malaria infection compared with no net use at all. This may be because these LLINs are treated with a pyrethroid, which can have non-lethal effects on mosquitoes that come into contact with it.

## Supplemental Materials

10.4269/ajtmh.25-0211 Supplemental Materials
