# Supplementary material for: Do Piperonyl Butoxide Long-Lasting Insecticide Treated Nets Provide Additional Protection Against Malaria Infections Compared with Conventional Nets in an Operational Setting in Western Kenya?
Source: Am J Trop Med Hyg. 2025 Nov 18;114(1):123–33. doi: 10.4269/ajtmh.25-0211 (PMC12781430; doi:10.4269/ajtmh.25-0211)
Supplement: Supplemental Materials [file tpmd250211.SD1.pdf]

SUPPLEMENTAL Materials

Supplemental Table S1. Monthly and Sick Visit responses of individuals who took an RDT (analytic population) versus all others who did not take an RDT during the study period

**Characteristic**	**Overall** *N* = 23,477[1]	**Took an RDT** *N* = 3,431[1]	**Did not take an RDT** *N* = 20,046[1]	***P*-Value**[2]
Slept Under a Net	16,602 (71%)	2,395 (69.8%)	14,207 (70.9%)	0.2
Net Washing Frequency				>0.9
At Least Monthly	1,272 (8.8%)	183 (8.8%)	1,089 (8.8%)	
Less Frequent	13,144 (91%)	1,905 (91.2%)	11,239 (91.2%)	
Unknown	9,061	1,343	7,718	
Net Type				<0.001
Non-PBO Net	11,963 (51%)	1,956 (57.0%)	10,007 (49.9%)	
Did Not Sleep Under Net	6,875 (29%)	1,036 (30.2%)	5,839 (29.1%)	
PBO Net	4,639 (20%)	439 (12.8%)	4,200 (21.0%)	
Gender				0.052
Female	12,848 (55%)	1,930 (56.3%)	10,918 (54.5%)	
Male	10,629 (45%)	1,501 (43.7%)	9,128 (45.5%)	
Age at Visit				<0.001
<5	2,780 (11.8%)	481 (14.0%)	2,299 (11.5%)	
5 to 15	9,308 (39.6%)	1,481 (43.2%)	7,827 (39.0%)	
>15	11,389 (48.5%)	1,469 (42.8%)	9,920 (49.5%)	

[1]n (%)

[2]Pearson's Chi-squared test

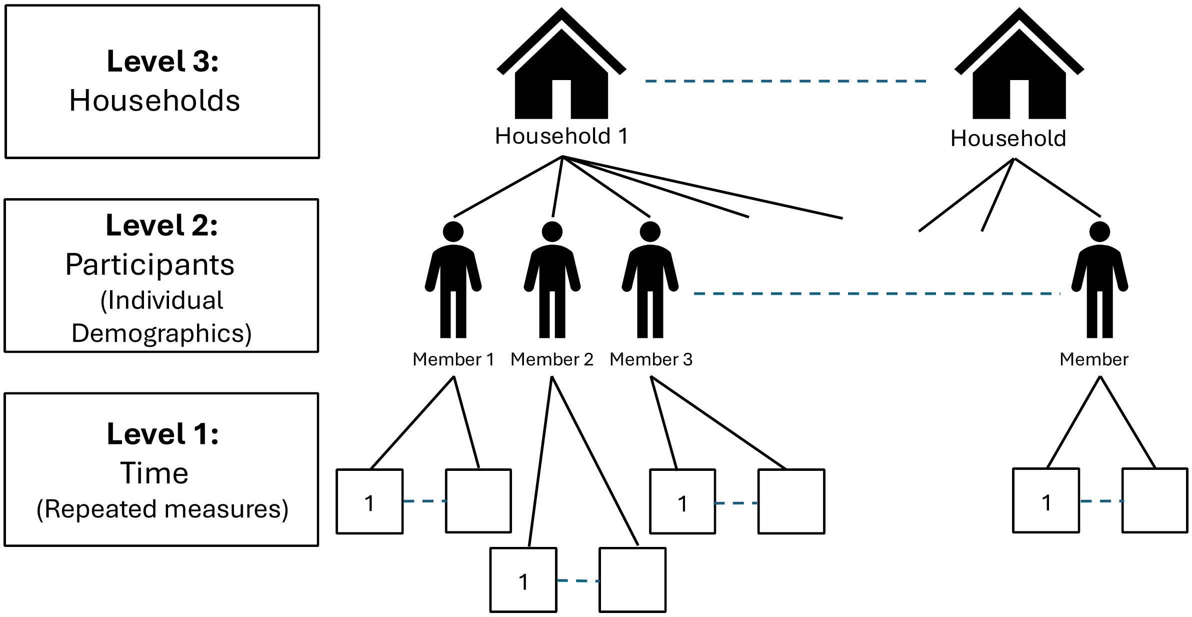

Supplemental Figure S1. Multilevel Model Hierarchical Structure Diagram.

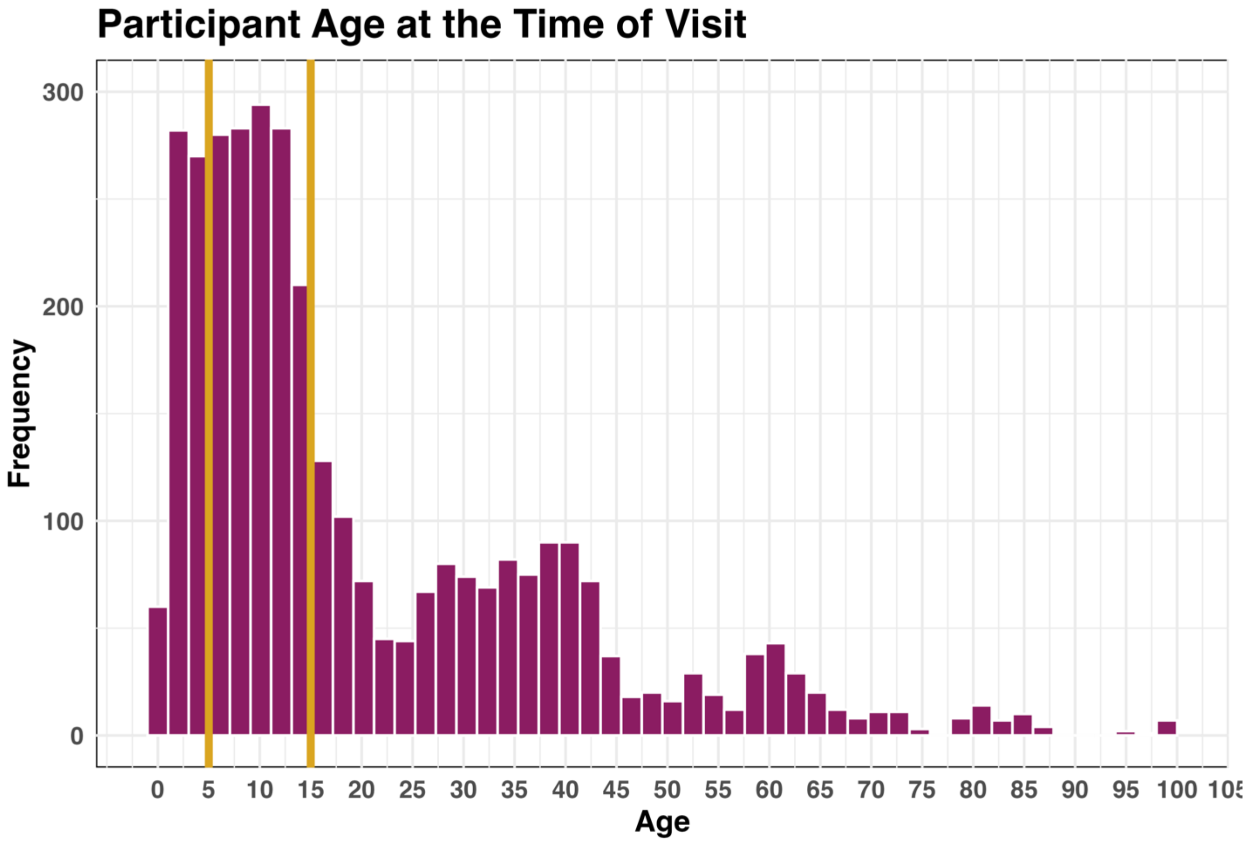

*Gold vertical lines denote age category cut-off points used in this study.

Supplemental Figure S2. Histogram distribution of study participant age at the time of visit.

Supplemental Table S2. Descriptive Statistics of main exposure (bed net use) across both the analytic and non-analytic population

	**Overall** N = 23,477[1]	**Conventional LLIN** N = 11,963[1]	**PBO Net** N = 4,639[1]	**Did Not Sleep Under Net** N = 6,875[1]
Took an RDT?				
Did not take an RDT	20,046 (85.4%)	10,007 (83.6%)	4,200 (90.5%)	5,839 (84.9%)
Took an RDT	3,431 (14.6%)	1,956 (16.4%)	439 (9.5%)	1,036 (15.1%)
Net Washing Frequency				
At Least Monthly	1,272 (8.8%)	876 (11.1%)	261 (6.5%)	135 (5.4%)
Less Frequent	13,144 (91.2%)	7,033 (88.9%)	3,737 (93.5%)	2,374 (94.6%)
Missing/NA	9,061	4,054	641	4,366
Village				
K	5,707 (24.3%)	3,687 (30.8%)	1,042 (22.5%)	978 (14.2%)
L	2,990 (12.7%)	1,611 (13.5%)	627 (13.5%)	752 (10.9%)
M	5,845 (24.9%)	3,103 (25.9%)	1,330 (28.7%)	1,412 (20.5%)
N	2,835 (12.1%)	914 (7.6%)	635 (13.7%)	1,286 (18.7%)
S	6,100 (26.0%)	2,648 (22.1%)	1,005 (21.7%)	2,447 (35.6%)
Gender				
Female	12,848 (54.7%)	6,979 (58.3%)	2,558 (55.1%)	3,311 (48.2%)
Male	10,629 (45.3%)	4,984 (41.7%)	2,081 (44.9%)	3,564 (51.8%)
Age at Monthly of Visit				
<5	2,780 (11.8%)	1,690 (14.1%)	647 (13.9%)	443 (6.4%)
5 to 15	9,308 (39.6%)	4,344 (36.3%)	1,571 (33.9%)	3,393 (49.4%)
>15	11,389 (48.5%)	5,929 (49.6%)	2,421 (52.2%)	3,039 (44.2%)
Net Age (in months)	21 (10, 33)	25 (13, 38)	14 (7, 21)	21 (11, 37)
Missing/NA	3,478	972	0	2,506
Net Holes				
Net had at least 1 hole	10,073 (54.4%)	6,167 (58.0%)	2,035 (45.1%)	1,871 (55.7%)
Net Intact	8,439 (45.6%)	4,470 (42.0%)	2,481 (54.9%)	1,488 (44.3%)
Missing/NA	4,965	1,326	123	3,516
Malaria Season				
Low Transmission Season	16,450 (70.1%)	8,280 (69.2%)	3,521 (75.9%)	4,649 (67.6%)
High Transmission Season	7,027 (29.9%)	3,683 (30.8%)	1,118 (24.1%)	2,226 (32.4%)

[1]person-months, n (%); Median (IQR)

Supplemental Table S3. Multicollinearity of Independent Variables

Term	VIF (95%CI)	SE_factor	Tolerance (95% CI)
Type of Net Used	1.663 (1.581, 1.756)	1.289	0.6014 (0.5694, 0.6325)
Study Year	1.621 (1.543, 1.712)	1.273	0.6168 (0.5843, 0.6482)
Village	1.136 (1.096, 1.194)	1.066	0.8800 (0.8378, 0.91240
Gender	1.031 (1.008, 1.113)	1.015	0.9704 (0.8988, 0.9918)
Categorical Age at Testing	1.070 (1.038, 1.129)	1.034	0.9348 (0.8859, 0.9636)
Net age in Months	1.360 (1.300, 1.430)	1.166	0.7356 (0.6992, 0.7690)
Net intact	1.073 (1.041, 1.132)	1.036	0.9318 (0.8836, 0.9610)
Transmission Season	1.023 (1.004, 1.128)	1.011	0.9777 (0.8869, 0.9959)

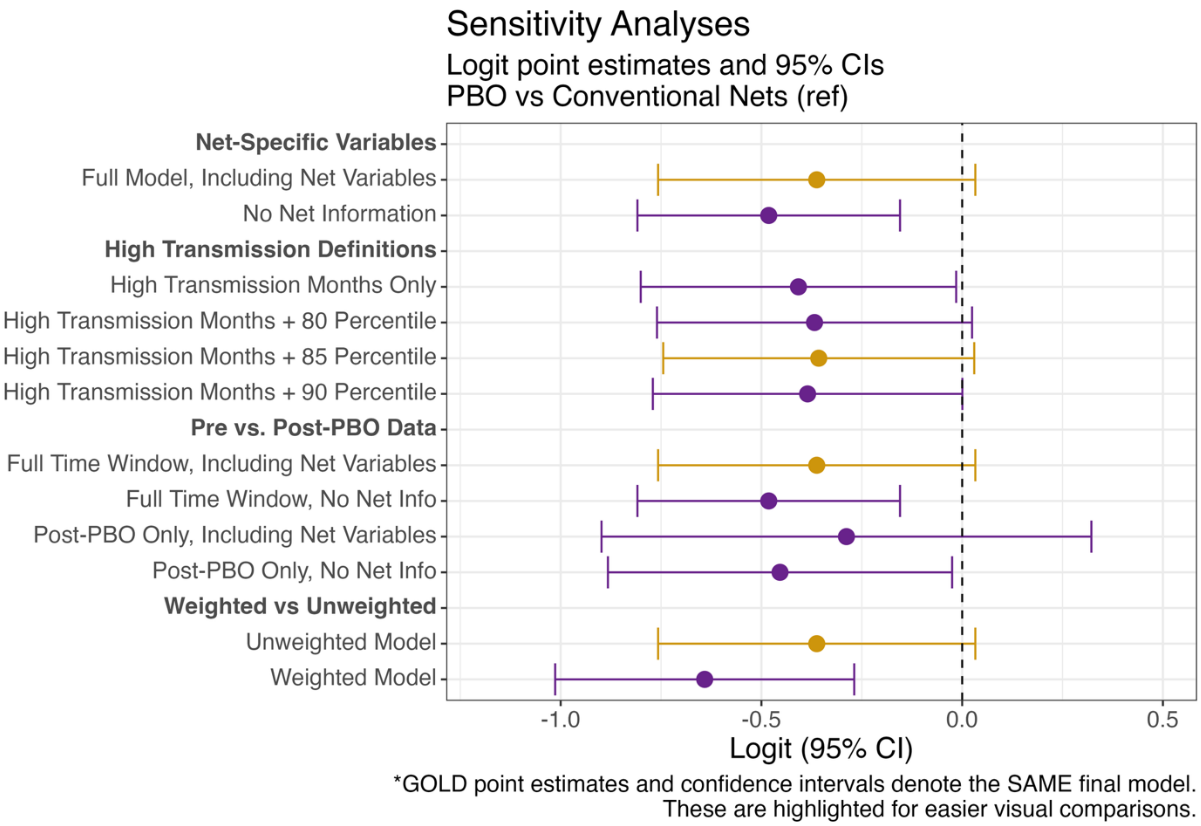

Supplemental Figure S3. Sensitivity analysis results, see *Supplementary Table S4* for model definitions.

Supplemental Table S4. Sensitivity Analyses, Model Definitions for Supplementary Figure S3

No.	Model Label	Definition
**1**	Full Model, Including Net Variables	Time window: June 2017- December 2023<br>Covariates:<br>• Type of net used<br>• Study Year<br>• Village<br>• Gender<br>• Categorical Age at Testing<br>• Net age in months<br>• Net intact (vs has at least 1 hole)<br>• Transmission season<br>• Based on monthly anopheles captures where 85th percentile values and above OR May/June/July months constitute high transmission months
**2**	No Net Information	Model 1 but *excludes*:<br>• Net age in months<br>• Net intact (vs has at least 1 hole)
**3**	High Transmission Months Only	Model 1 but replaces Transmission Season covariate with a fixed May/June/July definition
**4**	High Transmission Months + 80 Percentile	Model 1 but replaces Transmission Season covariate with a 80th percentile values
**5**	High Transmission Months + 85 Percentile	Same as Model 1
**6**	High Transmission Months + 90 Percentile	Model 1 but replaces Transmission Season covariate with a 90th percentile values
**7**	Full Time Window, Including Net Variables	Same as Model 1
**8**	Full Time Window, No Net Info	Same as Model 2
**9**	Post-PBO Only, Including Net Variables	Same as Model 1 but only looks at the post-PBO net distribution time window of June 2021 – Dec. 2023
**10**	Post-PBO Only, No Net Info	Same as Model 2 but only looks at the post-PBO net distribution time window of June 2021 – Dec. 2023
**11**	Unweighted Model	Same as Model 1
**12**	Weighted Model	Full model, with added weights to adjust for possible under-sampling of PBO net use, see Table S1

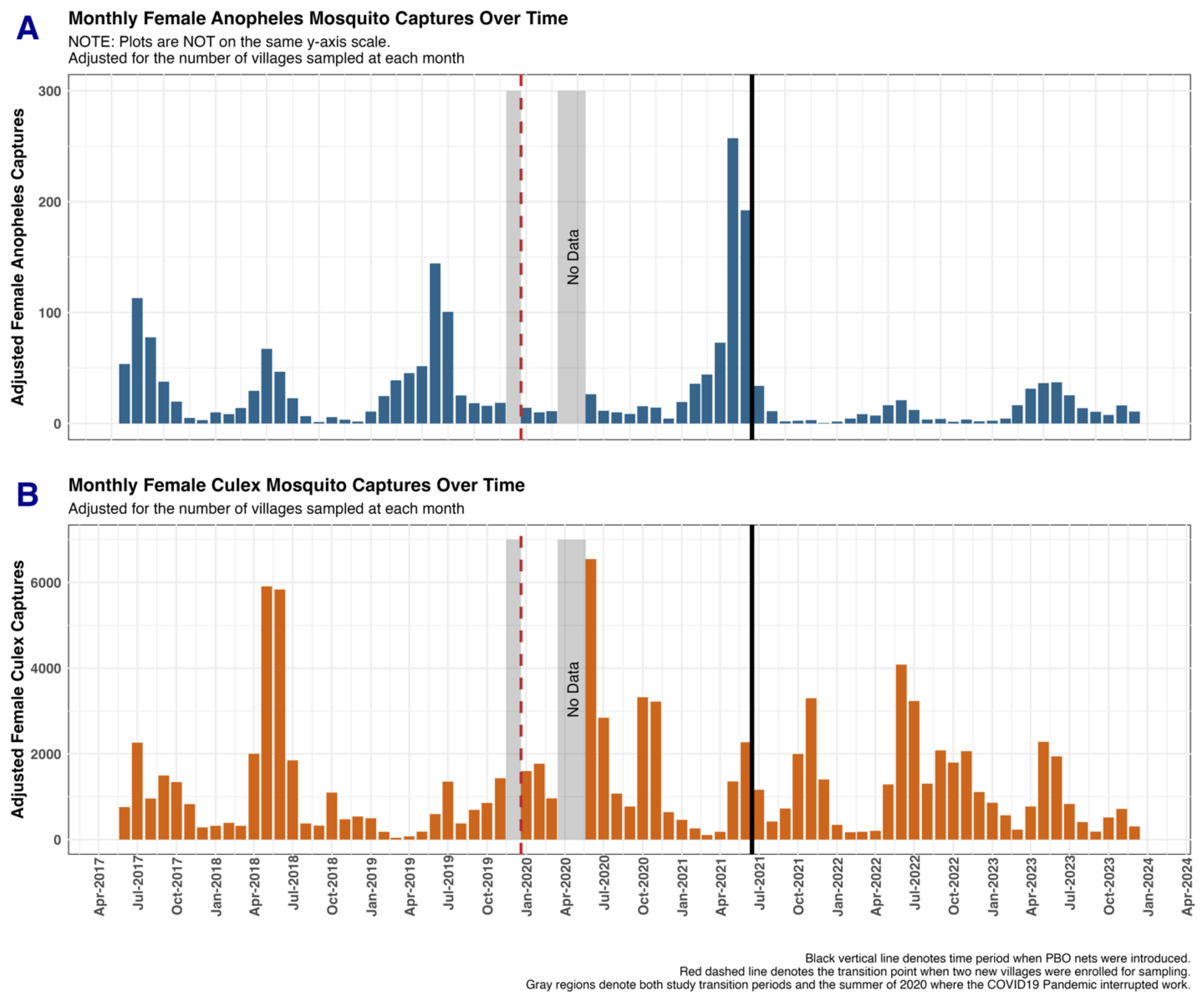

Supplemental Figure S4. Time series description of mosquito captures, adjusted for the number of villages sampled each month. Panel (A) describes female anopheles captures while (B) denotes female culex captures.

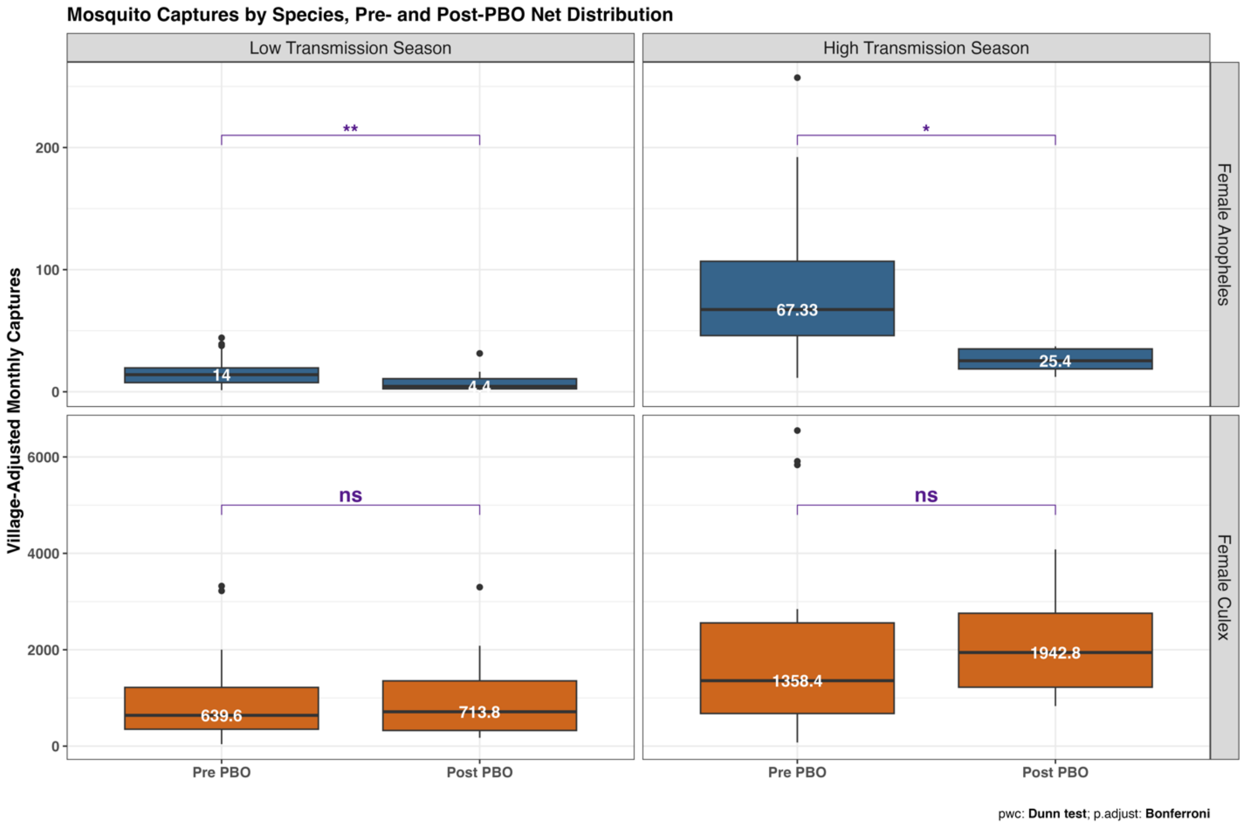

Supplemental Figure S5. Female mosquito captures pre- and post-PBO distribution, stratified by mosquito species and low versus high malaria transmission seasons.

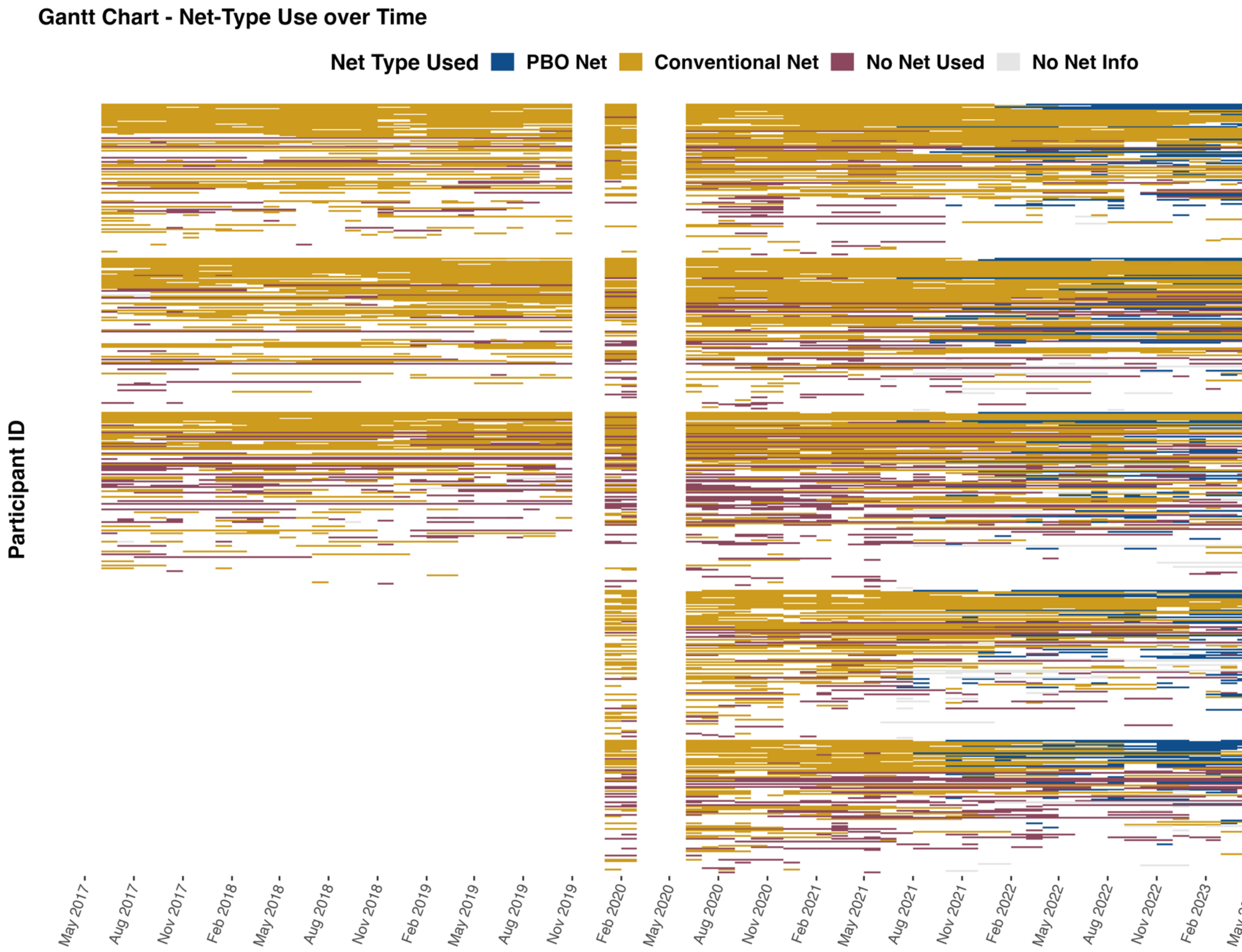

Supplemental Figure S6. Gantt chart of net-use over time. Observations are sorted in descending order by net-use where PBO net and conventional net use hold a “higher rank” and are consequently organized towards the top of each group, and no net use and no/missing information hold “lower ranks.” Records are further grouped by village from Village K (first from the top), Village M (second), Village S (third), Village L (fourth), and Village N (final bottom group).

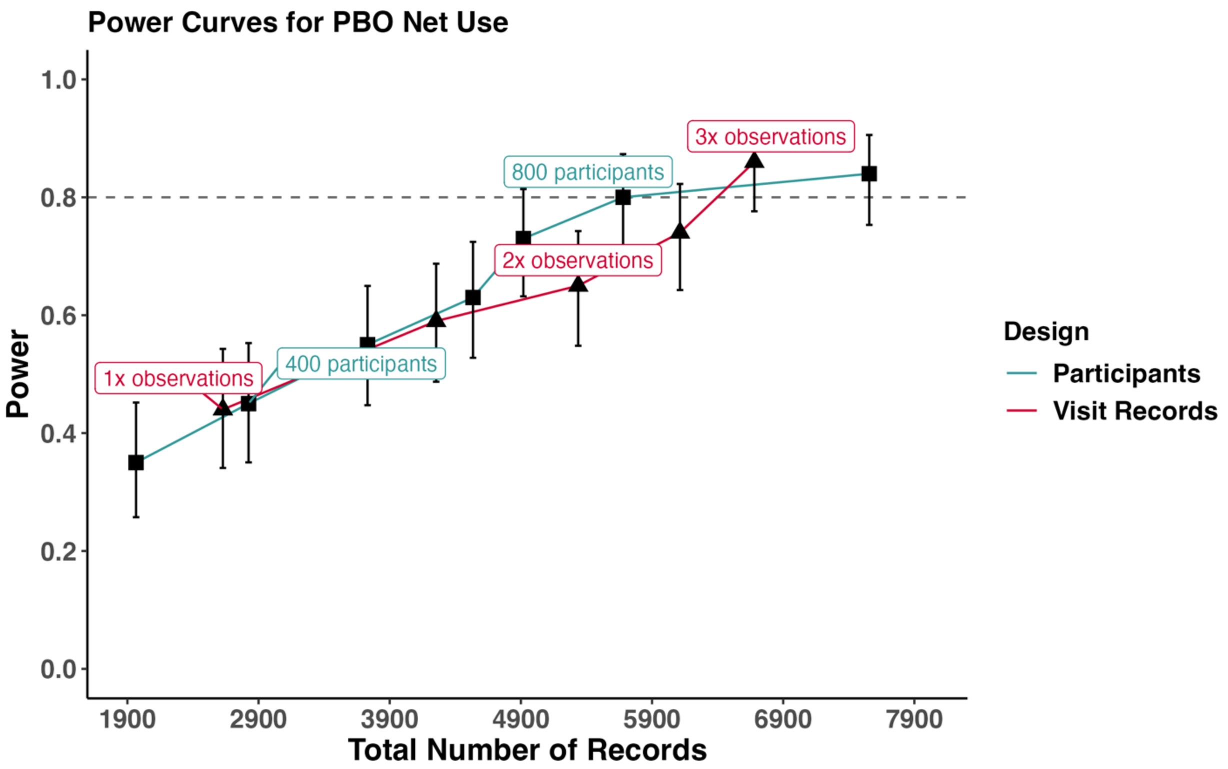

Supplemental Figure S7. Line plot illustrating the estimated study power associated with the varying number of participant sample size (blue line) and visit records (red line). Labels on this figure represent the minimum values necessary to achieve a study power of 80% for each, number of visit records and participant sample size.
